# An Immune-Independent Mode of Action of Tacrolimus in Promoting Human Extravillous Trophoblast Migration Involves Intracellular Calcium Release and F-Actin Cytoskeletal Reorganization

**DOI:** 10.3390/ijms252212090

**Published:** 2024-11-11

**Authors:** Ahmad J. H. Albaghdadi, Wei Xu, Frederick W. K. Kan

**Affiliations:** Department of Biomedical and Molecular Sciences, Faculty of Health Sciences, Queen’s University, Kingston, ON K7L 3N6, Canada; a.albaghdadi@queensu.ca (A.J.H.A.); wx@queensu.ca (W.X.)

**Keywords:** tacrolimus (TAC; FK506), extravillous trophoblasts, inositol triphosphate receptor (IP3R), FKBP12, [Ca^2+^]i, F-actin cytoskeleton

## Abstract

We have previously reported that the calcineurin inhibitor macrolide immunosuppressant Tacrolimus (TAC, FK506) can promote the migration and invasion of the human-derived extravillous trophoblast cells conducive to preventing implantation failure in immune-complicated gestations manifesting recurrent implantation failure. Although the exact mode of action of TAC in promoting implantation has yet to be elucidated, the integral association of its binding protein FKBP12 with the inositol triphosphate receptor (IP3R) regulated intracellular calcium [Ca^2+^]i channels in the endoplasmic reticulum (ER), suggesting that TAC can mediate its action through ER release of [Ca^2+^]i. Using the immortalized human-derived first-trimester extravillous trophoblast cells HTR8/SVneo, our data indicated that TAC can increase [Ca^2+^]I, as measured by fluorescent live-cell imaging using Fluo-4. Concomitantly, the treatment of HTR8/SVneo with TAC resulted in a major dynamic reorganization in the actin cytoskeleton, favoring a predominant distribution of cortical F-actin networks in these trophoblasts. Notably, the findings that TAC was unable to recover [Ca^2+^]i in the presence of the IP3R inhibitor 2-APB indicate that this receptor may play a crucial role in the mechanism of action of TAC. Taken together, our results suggest that TAC has the potential to influence trophoblast migration through downstream [Ca^2+^]i-mediated intracellular events and mechanisms involved in trophoblast migration, such as F-actin redistribution. Further research into the mono-therapeutic use of TAC in promoting trophoblast growth and differentiation in clinical settings of assisted reproduction is warranted.

## 1. Introduction

Tacrolimus (FK506 or TAC) is an immunosuppressive drug that is primarily prescribed for its calcineurin inhibitory actions [1] to reduce the risk of organ rejection among organ transplant recipients [2]. In 2011, our pre-clinical studies in mouse models of immunologically challenged gestations established, for the first time, a promising benefit for the use of TAC in preventing implantation failure [3,4,5]. Since then, there has been an increasing number of studies reporting a promising use for TAC as an alternative therapeutic option in treating recurrent implantation failure (RIF)/recurrent pregnancy loss (RPL) in infertile women, who are candidates of euploid embryo transfer after preimplantation genetic screening (PGS) [6,7,8]. Our perception of the mode of action of TAC was that it promoted a favorable immunological milieu for the implanting embryo and conditioned an immunologically tolerant uterine decidual response through its immunosuppressive properties [9,10]. However, further research from our group revealed a potential immunologically independent mode of action of TAC, presumably through promoting the migration and invasion of the first-trimester extravillous trophoblast (EVT) cells [11]. We thought that the demonstrated influence of TAC on the molecular mechanisms associated with progesterone receptor signaling highlights the immune-independent actions of TAC in supporting embryo implantation [11]. However, our latest research findings revealed that TAC has an additional and unique mode of action depicted in its nitric oxide synthase (eNOS)-modulatory actions stimulating the production and release of nitric oxide (NO) through triggering the phosphorylation of the eNOS active domain “p-eNOS-Ser1197” as well as STAT3-Y705 conducive to promoting the migration and invasion of the first human trimester EVT cells cultured under nitrosative stresses in vitro [9].

Collectively, our pre-clinical data suggested that the efficacy of TAC in mitigating the severity and incidence of implantation failure and restoring proper spiral artery remodeling in fetal growth-restricted gestations involves an immune-independent mode of action of TAC that needs to be clinically considered for the prevention of RIF/RPL in women [5,11]. Therefore, to provide further insights into these plausible immune-independent actions of TAC in treating RIF/RPL, this study reports yet intriguing findings of distinctive cellular mechanisms of TAC, in promoting the migration of human EVT cells through its influence on their intracellular calcium [Ca^2+^]-release and the associated dynamic and structural reorganization of their F-actin cytoskeleton. Actin is an essential cytoskeletal protein crucial for cell survival [12]. In its filamentous form, actin provides tracks for the movement of intracellular materials and a driving force for cell movements [12,13]. Under physiological conditions, actin monomers spontaneously polymerize into long, stable filaments with a helical arrangement of subunits [14]. This process is highly responsive to [Ca^2+^]i and the polymerized actin filaments will subsequently distribute throughout the cells in a dynamic state of transitions between a cortical- and a stress-fiber-dominated organization of intracellular actin networks [15].

Although the present research aimed to elucidate the molecular mechanisms and cellular pathways involved in mediating TAC immune-independent actions in promoting embryo implantation, the integral association of its binding protein FKBP12 and the IP3R-regulated intracellular calcium [Ca^2+^]i channels in the endoplasmic reticulum (ER) suggest that TAC can mediate its cellular action through the ER release of [Ca^2+^]i [10,16]. The inositol triphosphate receptor (IP3R) is an intracellular Ca^2+^ release channel located on the membrane of the ER and belongs to the same family as the ryanodine receptors (RyRs) [16,17]. Here, we hypothesized that TAC enhances trophoblast cell migration by promoting the release of intracellular calcium stores through the IP3R pathway and influencing the F-actin structural organization conducive to enhancing trophoblast cell migration. The specific objectives of the present research are: (1) to determine if TAC influences [Ca^2+^]i release in trophoblast cells; (2) to determine if the TAC-influenced [Ca^2+^]i release in trophoblast cells is IP3R-depenent; and (3) to determine if the TAC-influenced [Ca^2+^]i release in trophoblast cells is associated with F-actin cytoskeletal reorganization.

## 2. Results

### 2.1. Tacrolimus Spiked the ER[Ca^2+^]i Release in Trophoblast Cells in an IP3R-Dependent Manner

**Figure 1:**
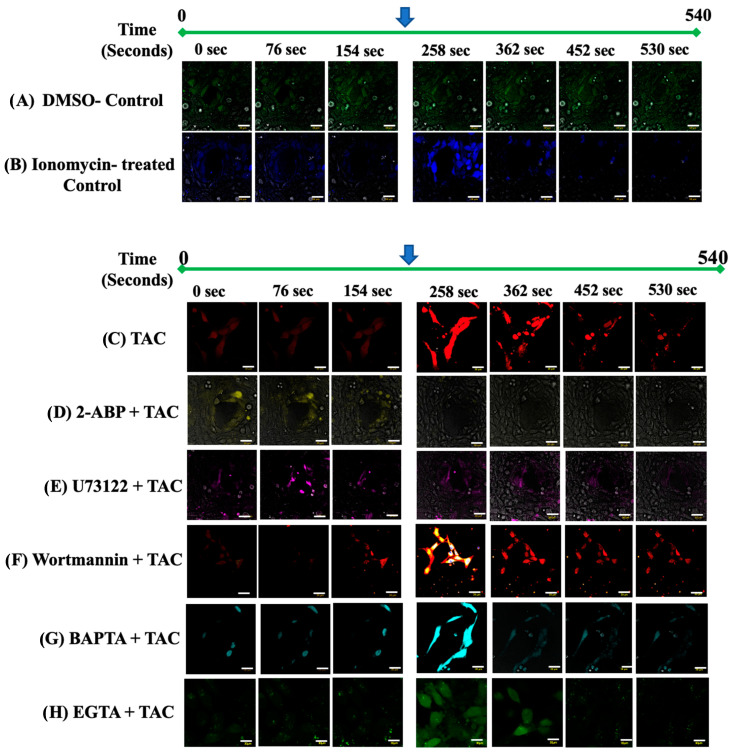
Tacrolimus spiked [Ca^2+^]i release in live HTR8/Svneo cells. (**A**–**H**): Representative photomicrographs of individual live HTR8/SVneo cells visualized by intravital confocal microscopy depicting the intracellular contents of Ca^2+^ in DMSO-treated control ((**A**): green), ionomycin ((**B**): blue), TAC-treated ((**C**): red), 2-APB + TAC ((**D**): yellow), U73122 + TAC ((**E**): purple), Wortmannin + TAC ((**F**): orange), BAPTA + TAC ((**G**): turquoise) and EGTA + TAC ((**H**): green), respectively. The timeframe for Ca^2+^ imaging was for a total of 540 s. [Ca^2+^]i release was spiked at the end of minute 3 of live-image tracing (depicted by the blue arrow) by the addition of ionomycin (**B**), or TAC alone (**C**) or in the presence of other inhibitors of [Ca^2+^]i release and Ca^2+^ chelators (**D**–**G**). Compared to the DMSO-treated (**A**) and Ionomycin-treated controls (**B**), the single use of TAC (10 ng/mL) resulted in a significant increase in [Ca^2+^]i in the HTR8/SVneo cells (compare the intensity of red color in (**C**) before and after the addition of TAC). The inability of TAC to spike [Ca^2+^]i-release in the presence of the IP3R antagonist 2-APB (**D**) suggests a crucial role for IP3R in mediating the [Ca^2+^]i modulatory actions of TAC. Unexpectedly, the TAC-induced [Ca^2+^]i-release in HTR8/SVneo cells was unaffected by inhibitory actions of the PLC inhibitor U73122 (**E**) as well as the potent and specific phosphatidylinositol 3-kinase (PI3-K) inhibitor Wortmannin (**F**). The intracellular source of the TAC-induced [Ca^2+^]i-release is confirmed by the use of intracellular [Ca^2+^]i chelator BPATA (**G**) and EGTA (**H**). Scale bars: (**A**–**H**) 30 µm; [Ca^2+^]i is depicted in pseudocolors in (**A**–**H**).

**Figure 2:**
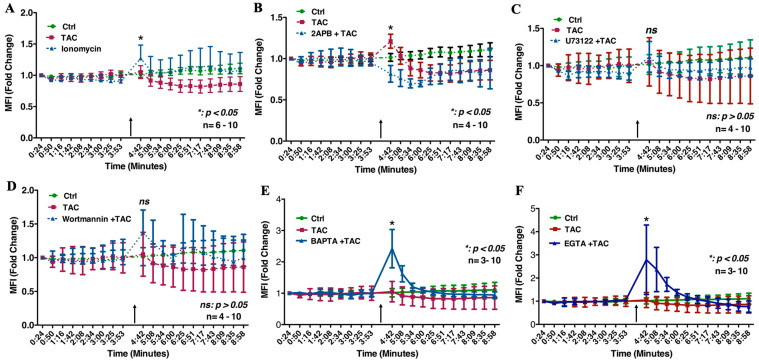
Real-time tracing of [Ca^2+^]i-release in live HTR8/SVneo cells. (**A**–**F**): Fold change in Mean Fluorescence Intensity (MFI) of Fluo-4 as a measurement of [Ca^2+^]i-release in HTR8/SVneo cells receiving TAC (**A**) in the presence and absence of the [Ca^2+^]i-release inhibitors (2-APB: (**B**), U73122: (**C**), Wortmannin: (**D**)), as well as the Ca^2+^chelators BAPTA (**E**), and EGTA (**F**). (**A**) depicts the influence of TAC administration on spiking [Ca^2+^]i-release (black arrow) in a comparable intensity to that of the calcium ionophore Ionomycin. (**B**) depicts pre-incubation with the IP3R inhibitor 2-APB significantly suppressed TAC-induced [Ca^2+^]i-release in HTR8/SVneo cells (*p* < 0.05). (**C**,**D**): Unlike the suppressive effects of the 2-APB on TAC-induced [Ca^2+^]i-release, the inhibition of the phospholipase C (PLC) or the phosphatidylinositol 3-kinase (PI3K) by use of the compound U73122 (**C**) and Wortmannin (**D**) did not restrict this cellular action of TAC. In (**E**,**F**), the spiked TAC-induced [Ca^2+^]i-release in HTR8/SVneo cells concomitantly treated with the intracellular and extracellular calcium chelators BAPTA (**E**) and EGTA (**F**) suggests the release of [Ca^2+^]i is from the intracellular stores, namely the endoplasmic reticulum. In (**A**–**F**), HTR8/SVneo cells were pre-incubated for 10 min with the [Ca^2+^]i-release inhibitors prior to the administration of TAC. Real-time tracing of [Ca^2+^]i-release in Ionomycin-treated HTR8/SVneo cells was included in (**A**) for a comparison. Black arrows in (**A**–**F**) depict spiked [Ca^2+^]i-release. The time recording was 10 min.

The crucial roles of the RYR channels in regulating the cell migration of the HTR8/SVneo cells and related cellular activities in other placental cell lines conducive to proper placental development and functionality have been previously reported [18,19,20]. Compared to the DMSO-treated (Figure 1A and Figure 2A) and ionomycin-treated controls (Figure 1B and Figure 2A), the solitary use of TAC (10 ng/mL) resulted in a significant increase in free [Ca^2+^]i in the HTR8/SVneo cells (Figure 1C and Figure 2A). To discern the involvement of the individual components of the IP3R [Ca^2+^]i-release pathway, we used the membrane-penetrable inhibitor of inositol(1,4,5)P3-induced Ca^2+^ release, namely the 2-Aminoethoxydiphenyl borate/2-Aminoethyl diphenyl borate (2-APB) [21,22], as well as the highly selective phospholipase C (PLC) inhibitor U-73122 which inhibits Ca^2+^ release from the intracellular sarcoplasmic reticulum Ca^2+^ store, and the specific phosphatidylinositol 3-kinase (PI3K) inhibitor Wortmannin. Moreover, to ascertain the source of the [Ca^2+^]i release originating from intracellular Ca^2+^ stores, namely the endoplasmic reticulum, we have also used the intracellular calcium chelators BAPTA and EGTA, respectively [20,23]. As depicted in Figure 1D and Figure 2B, the ability of TAC to spike [Ca^2+^]i-release was lost in the presence of 2-APB thus annotating the presence of a functional Inositol-(1,4,5)-P3 as a prerequisite for the [Ca^2+^]i-release action of TAC. This also suggests that IP3R-mediated [Ca^2+^]i release is likely a feature of TAC tacrolimus’ immunosuppressant-independent mechanism of action in influencing actin cytoskeletal redistribution in trophoblast cells during migration and invasion. On the other hand, TAC was unexpectedly found to be capable of spiking [Ca^2+^]i-release in the presence of functional PLC and PI3K inhibition mediated by U73122 (Figure 1E and Figure 2C) and Wortmannin (Figure 1F and Figure 2D), respectively. Lastly, our data indicate that the source of [Ca^2+^]i-release in TAC-treated HTR8/SVneo cells originated at intracellular Ca^2+^ stores, particularly the ER, as demonstrated by the significant spike in [Ca^2+^]i release when these cells were pre-treated with BAPTA and EGTA (Figure 1G,H and Figure 2E,F, respectively). Taken together, it is apparent that whereas the IP3R may be critically involved in mediating the [Ca^2+^]i-release actions of TAC leading to enhanced trophoblast migration, the TAC-mediated reversal of suppressed [Ca^2+^]i-release in the Wortmannin- and U73122-treated cells may suggest weak chemical affinity of these inhibitors to bind their intended targets along the IP3R [Ca^2+^]i-release pathway in the presence of TAC. Therefore, based on these findings, it is challenging to confirm whether PI3K may serve as a target for TAC that is pertinent to the IP3R [Ca^2+^]i-release mechanism. Nonetheless, the reported PLC inhibitory activity of BAPTA independent of their Ca^2+^ chelating properties [20] lends further support to the observed significant role of ER-associated components of the RYR in the presently reported actions of TAC in favorably influencing ER [Ca^2+^]i-release conducive to promoting trophoblast cell migration in vitro.

### 2.2. TAC-Influenced ER[Ca^2+^]i Release in Trophoblast Cells Is Associated with F-Actin Cytoskeletal Reorganization

**Figure 3:**
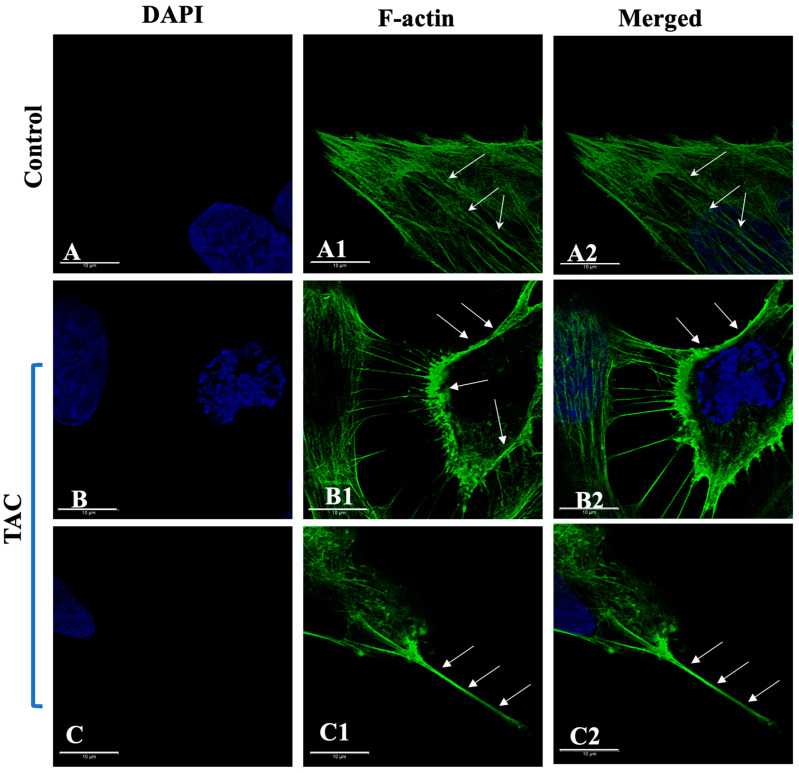
Tacrolimus influences F-actin cytoskeletal re-arrangement in the human-derived first-trimester extravillous trophoblast cells. (**A**–**C**): Single cell confocal images of control (**A**) and TAC-treated HTR8/SVneo cells (**B**,**C**) labeled with the CellMask Green^TM^ Actin tracking stain. F-actin is mostly distributed in the form of stress fibers running across the cell body of untreated cells (white arrows in (**A1**,**A2**)). 10 min pre-incubation with TAC resulted in a global reorganization of the F-actin filaments manifested in the formation of cortical fibers (white arrows in (**B1**,**B2**)). Notably, filopodia-like structures (white arrows in (**C1**,**C2**)) were observed among TAC-treated HTR8/SVneo cells evidently demonstrating a tangible outcome of the influence of TAC on F-actin cytoskeletal reorganization suggestive of cell motility. Green: CellMask Green^TM^-labled F-actin, Blue: DAPI-stained nuclei. Scale bars: (**A**–**C**) 10 µm. Nuclei were counterstained with DAPI in (**A**–**C**).

**Figure 4:**
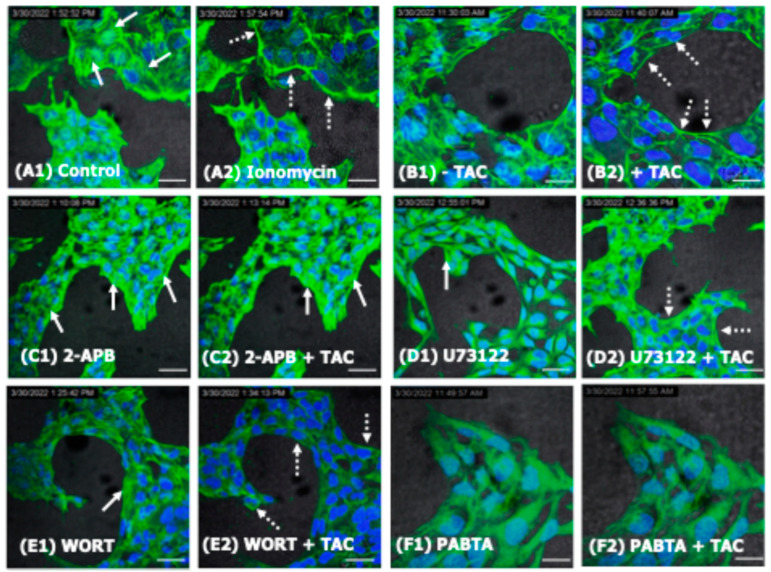
The influence of [Ca^2+^]i-release inhibitors and chelators on the structural distribution of F-actin in TAC-treated HTR8/SVneo cells. (**A**–**F**): Time-dependent cytoskeletal reorganization of F-actin in the HTR8/SVneo cells in response to Ionomycin (**A1**,**A2**), TAC (**B1**,**B2**), 2-APB (**C1**,**C2**), U73122 (**D1**,**D2**), Wortmannin (**E1**,**E2**) and PABTA (**F1**,**F2**), respectively. Note the characteristic distribution of the stress fibers throughout the cytoplasm (solid white arrows) versus the peripherally condensed cortical fiber (dashed white arrows). Failure of F-actin cellular reorganization in the 2-APB-inhibited cells ((**C1**) vs. (**C2**)) indicates the dependence of TAC actions on the functional IP3R-signaling pathway. Distinctly, unlike pre-incubation with U73122 (**D1**,**D2**) and Wortmannin (**E1**,**E2**), the presence of 2-APB (**C1**,**C2**) and PABTA (**F1**,**F2**) compromised the structural integrity and consequently the visualization of the F-actin cytoskeleton in HTR8/SVneo cells. The recording after the addition of TAC to the inhibitor pre-treated cells was 6 min. Green: CellMask Green^TM^-labled F-actin, Blue: DAPI-stained nuclei. Scale bars: (**A1**–**F2**) 45 µm.

The influence of TAC on the structural reorganization of the actin cytoskeleton in HTR8/SVneo cells was recorded in real time using control and treated live HTR8/SVneo cells and the cell-permeable F-actin marker CellMask Green^TM^ actin tracking stain. As depicted in Figure 3, as opposed to the prominent distribution of F-actin stress fibers in the untreated HTR8/SVneo cells (Figure 3(A1,A2)), the foremost action of the single use of TAC is a major structural reorganization of F-actin cytoskeleton predominantly characterized by the formation of cortical fibers in these cells (Figure 3(B1,B2)). Notably, this core structural rearrangement of the F-actin cytoskeleton in HTR8/SVneo cells was associated with the formation of filopodia-like membrane protrusions within 10 min of the administration of TAC (Figure 3(C1,C2)). Moreover, comparable to the actions of the calcium ionophore ionomycin (Figure 4(A1,A2)), a time-dependent cytoskeletal reorganization of F-actin was also successfully induced and visualized in the HTR8/SVneo cells in response to TAC (Figure 3(B1,B2)). Remarkably, as seen in Figure 4(A1,A2,B1,B2), both treatment conditions favored a predominant cortical F-actin redistribution in HTR8/SVneo cells. However, unlike the pre-incubation with U73122 (Figure 4(D1,D2)) and Wortmannin (Figure 4(E1,E2)), the presence of 2-APB (Figure 4(C1,C2)) and PABTA (Figure 4(F1,F2)) was detrimental for the detection of F-actin cellular reorganization in these trophoblast cells and dictated a dependence of TAC actions on functional IP3R-signaling pathway in inducing F-actin cytoskeletal reorganization. The recording after the addition of TAC to the inhibitor pre-treated cells was 6 min.

### 2.3. TAC-Influenced ER[Ca^2+^]i Release in Trophoblast Cells Is Associated with Reduced Co-Localization of IP3R and FKBP12 in HTR8/SVneo Cells

**Figure 5:**
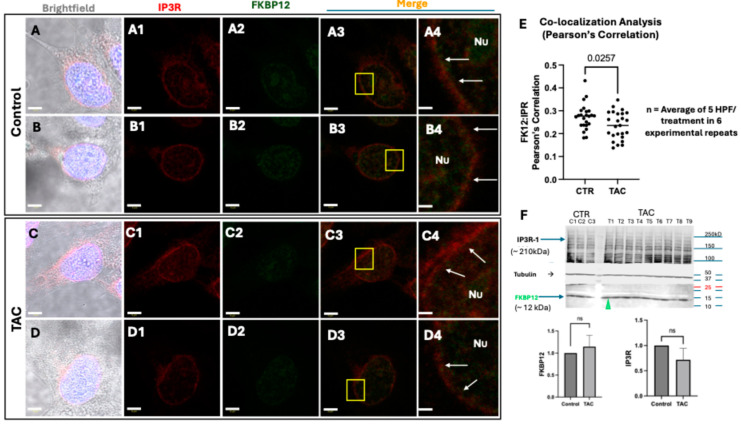
Tacrolimus negatively influenced the co-localization of IP3R and FKBP12 in HTR8/SVneo cells. (**A**–**E**): Representative confocal images for the immunofluorescent detection of IP3R-1 ((**A1**–**D1**): red *) and FKBP12 ((**A2**–**D2**): green *) and their co-localization ((**A3**–**D4**); firey orange, and co-localization analysis bar graphs in (**E**)) in PFA-fixed monolayers of control (DMSO-treated) and TAC-treated HTR8/SVneo cells depicting a significant reduction (*p* = 0.025) in the Pearson’s correlation coefficient of mean fluorescent intensities (MFI) of the co-localization of these two protein components of the ER [Ca^2+^]i-release channels after 1 h of exposure to TAC (**E**). Indeed, Pearson’s correlation quantification revealed that IP3R-I and FKBP12 are co-expressed in the same pixel in control cells more than in TAC-treated cells **. Note that the characteristic perinuclear distribution of these two proteins in HTR8/SVeno cells (white arrows in (**A4**–**D4**), respectively). (**F**): representative Western blot (Wb) detection of IP3R and FKBP12 in control (experimental repeats C1–C3 in lanes C1–C3) and TAC-treated (experimental repeats T1–T9 in lanes T1–T9) HTR8/SVneo cells, revealing a preservative effect of TAC in maintaining the levels of these two protein components of the ER [Ca^2+^]i-release channels in trophoblasts as measured by the lack of a significant fold change in their protein band intensities compared to untreated control cells (*p* > 0.05). The IP3R-1 bar graphs in (**F**) exclusively depict the intensities of the Wb protein bands at the 210 kDa molecular weight. Images in (**A4**–**D4**) are higher magnifications of the yellow-boxed cellular areas in (**A3**–**D3**), demonstrating the peri-nuclear distribution and co-localization of IP3R-1 and FKBP12 in control and TAC-treated HTR8/SVneo cells, respectively. (**A**,**B**): Representative brightfield images of control and TAC-treated HTR8/SVEneo cells depicting their general morphology and their blue-colored DAPI-stained nuclei, respectively. Scale bars: (**A**–**D4**) 5 µm. Nu (**A4**–**D4**): Nuclei. TAC: Tacrolimus. ns in (**F**): Not statistically significant (*p* > 0.05). *: Alexa-Fluor 790-conjugated anti-FKBP12 (anti-FKBP12-AF790; mouse anti-human) and Alexa-Fluor 680-conjugated IP3R-I (anti-IP3R-1-AF680; mouse anti-human) primary antibodies were used for the detection of their respective proteins labeled in the confocal images of HTR8/SVneo cells shown in (**A1**–**D4**). Due to current technical limitations with the wavelength detection of the 790 fluorochrome, anti-FKBP12-AF790-labeled monolayers of these cells were allowed a brief incubation with FITC-labeled goat-anti-mouse antibody suspension prior to re-incubation with the anti-IP3R-1-AF680 as described in the methods section. **: The quantification was performed by comparing all the individual frames (one cell per frame; four cells per experiment; a total of six experiments; therefore, 24 cells per treatment). Scatter blots in (**E**) represent the average rate of four cells imaged in randomly selected 5 high-power fields (HPFs) in an experiment (n = 6 plates (30 mm) per treatment).

Based on the established concept that FK506 Binding Protein 12 and its related isoform 12.6 (FKBP12/12.6) stabilizes a closed state of the ryanodine receptors [RyRs] conducive to controlling their [Ca^2+^]i release [24] and the reported disruptive influence of TAC on the functional and structural stability of the FKBP12/12.6/RYRs complex through binding and displacing FKBP12/12.6 from RyRs [24], we investigated a plausible immune-independent action of TAC on ER [Ca^2+^]i release via its binding to the FKPB12. We performed co-localization studies of these two proteins in TAC-treated HTR8/SVneo cells. As depicted in Figure 5(A1–B4,C1–D4), contrary to the DMSO-treated control (Figure 5(A1–B4)), a short 1 h treatment with TAC (Figure 5(C1–D4)) resulted in a significant reduction in the co-localization intensities of these proteins as demonstrated by the visible decrease in the yellow fluorescence in the merged sections (Figure 5(A1–B4) versus Figure 5(C1–D4)). This observation was further reinforced through a significant reduction in the Pearson’s Correlation coefficient of the measured values of their mean fluorescence intensities (MFI), demonstrating a detrimental influence of TAC on the affinity binding of these two protein components of the ER[Ca^2+^]i release channels in human trophoblast cells as shown in Figure 5E. Notably, the brief 1 h treatment with TAC did not seem to have influenced the protein expression of IP3R-1 and FKBP12 in treated HTR8/SVneo cells, as indicated by the lack of significance of the Western blot band intensities of these two proteins depicted in Figure 5F. This lends support to the notion that the ER[Ca^2+^]i release actions of TAC in HTR8/SVneo cells are likely functional and that ER[Ca^2+^]i release actions of TAC may not necessarily involve protein translational activities in these cells.

## 3. Discussion

In the present study, we have provided additional evidence for the immune-independent actions of TAC in promoting trophoblast cell migration and invasion. The results obtained in this work are consistent with our previous findings [9,10], supporting direct cellular actions of TAC on the actin cytoskeletal organization in trophoblast cells, conducive to their stimulated migratory and invasive capabilities. The focus of this study was to investigate the putative role of TAC in influencing the dynamic distribution of the cellular actin network through an inositol triphosphate receptor (IP3R)/[Ca^2+^]i-dependent mechanism. Ca^2+^ is a unanimous intracellular second messenger involved in a multitude of cellular processes such as signal transduction, enzyme and hormone secretion, cell cycle regulation, and programmed cell death. Notably, Ca^2+^ plays a critical role in the process of cellular morphogenesis involved in cell motility, and [Ca^2+^]i increase is known to induce polymerization of the actin cytoskeleton in a variety of cellular contexts [25]. The actin cytoskeleton is homeostatic, and its organization and dynamics are critical for most morphogenetic processes, including cell polarization, migration, and division [26,27]. The conspicuous role of the actin cytoskeleton is imitated in a host of regulators that mediate the dynamic assembly of complex actin structures from filament bundles and networks, which are involved in the provision of protrusive and contractile forces during physiological and pathological processes such as migration, ECM-degradation, wound healing, and tumor metastasis [27,28]. This cellular response to [Ca^2+^]i influx, referred to as calcium-mediated actin reset (CaAR), as first described by Wales et al. [29], represents a unique calcium ion-mediated cellular mechanism which is involved in deconstructing large parts of the actin cortex and simultaneously forming actin filaments near the center of the cell [29]. CaAR is a short-lived transient actin cytoskeletal rearrangement that lasts for only a few minutes before the actin cortex is reorganized, forming new filaments near the endoplasmic reticulum through the concerted actions of distinct actin nucleators that are constantly competing for a common pool of actin monomers [30]. Moreover, a previous study by Shao et al. (2015) [31] reported the rapid and transient formation of calcium-mediated actin filaments at the nuclear periphery of fibroblasts exposed to mechanical stress [31]. These observations are also supported by reports revealing the central role of [Ca^2+^]i influx in promoting the polymerization of linear actin filaments emanating from the inner nuclear membrane toward the nuclear interior, resulting in nuclear actin-dependent alterations in chromatin organization and the fostering of nuclear F-actin assembly for rapid responses toward chromatin dynamics. In fact, several reports suggest a central role for dynamic nuclear F-actin assembly in the regulation of nuclear volume expansion, chromatin de-condensation, and gene transcription, as well as homology-directed DNA repair [30,32,33,34,35]. Taken together, experimental evidence presented in the present study suggests that TAC tacrolimus can critically influence the dynamic reorganization of actin filaments in the cytoplasm for mechanical support, membrane dynamics, and intracellular trafficking in trophoblast cells. These data lend support to our previous observations, which suggest that TAC tacrolimus might have influenced a wide-scale gene expression in trophoblast cells conducive to their enhanced migratory behaviors independent of its immune-suppressive capabilities [9,11].

### 3.1. Tacrolimus-Induced [Ca^2+^]i Release Requires Functional Inositol Triphosphate Receptor (IP3R)

**Figure 6:**
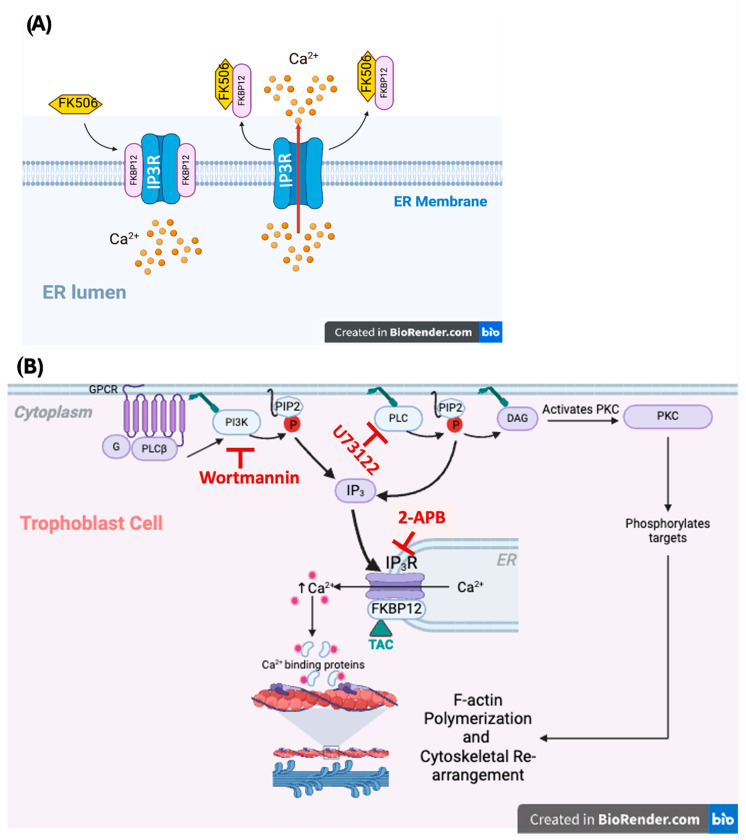
(**A**): Schematic depicting of the inositol triphosphate receptor (IP3R), which is an intracellular Ca^2+^-release channel located on the membrane of the endoplasmic reticulum (ER), and which belongs to the same family of the ryanodine receptors (RyRs). The conserved and widely abundant immunophilin FKBP12, which is a primary receptor for the immunosuppressant actions of TAC (FK506), has been demonstrated to physiologically interact with the inositol 1,4,5-trisphosphate receptor (IP3R) via a leucyl-prolyl dipeptide epitope that structurally resembles TAC (FK506). Here, we are postulating that TAC binding to FKBP12, likely through its structural mimicry to dipeptide epitopes on the FKBP12, sequesters this immunophilin from the IP3R, thus structurally destabilizing the channel conducive to a spiked release of [Ca^2+^]i from ER stores (arrow). Abbreviations: TAC (FK506): tacrolimus; IP3R: inositol triphosphate receptor; ER: endoplasmic reticulum. (**B**): Schematic depicting of the inositol triphosphate receptor (IP3R) [Ca^2+^]i-release pathway in trophoblast cells. The illustration depicts a potential mechanism through which TAC may influence [Ca^2+^]i-release along the IP3R pathway and its putative intracellular signal transduction pathways involved in F-actin cytoskeletal reorganization in trophoblast cells. [Ca^2+^]i-release in trophoblasts is normally a function of the G-protein-coupled receptor (GPCR)-mediated activation of phospholipase C (PLC) and the membrane-bound PI3K (which produces inositol triphosphate (IP3)). IP3 is a ligand for the intracellular IP3R channel of the internal Ca^2+^ stores of the endoplasmic reticulum (ER). It is postulated that TAC influences [Ca^2+^]i-release via its binding to the immunophilin FKBP12, plausibly resulting in the destabilization of the ER’s IP3R [Ca^2+^]i-release channels. The observation that TAC was unable to release [Ca^2+^]i in trophoblast cells in the presence of the IP3R inhibitor 2-APB suggests a major role for this RYR channel in the presently proposed mode of action of TAC. This notion is also supported by the ability of TAC to release [Ca^2+^]i in the presence of the PI3K inhibitor Wortmannin, and the PLC inhibitor U73122. Moreover, PLC activation can also lead to the production of diacylglycerol (DAG), which in turn activates protein kinase C (PKC), contributing to F-actin polymerization through the phosphorylation of a large library of intermediate targets of Ca^2+^ binding proteins. Based on data obtained in the present study, it is presently unclear if TAC-induced [Ca^2+^]i-release can influence the activation of a multitude of Ca^2+^-binding proteins involved in the F-actin polymerization through the PKC signaling pathway. Abbreviations: TAC (FK506): tacrolimus; GPCR: G-coupled protein receptor; PLC: phospholipase C; IP3: inositol (1,4,5)3-phosphate; IP3R: inositol triphosphate receptor; PI3K: phosphatidylinositol 3-kinase; PKC: protein kinase C.

The inositol triphosphate receptor (IP3R) is an intracellular Ca^2+^ release channel belonging to the ryanodine receptor (RYR) superfamily, which is positioned on the membrane of the endoplasmic reticulum, as illustrated in Figure 6A. Previous studies highlighted the requirement for functional IP3R channels for normal placental development. A report by Uchida et al. (2016) [36] revealed defective placental development in an IP3R knockout murine model due to poor trophoblast cell migration [36]. Remarkably, a recent report by Zheng et al. (2022) [19] confirmed the pre-requisite of F-actin cytoskeletal reorganization in the IP3R-mediated regulation of trophoblast cell migration [19]. The binding of the immunophilins FK506 binding proteins, 12 and 12.6 (FKBP12/12.6), to the ryanodine receptors [RyRs] is reported to stabilize a closed state of their [Ca^2+^]i-release activity [37,38,39,40,41]. Indeed, it has been shown in a variety of cellular contexts that the displacement of FKBP12/12.6 from RyRs or mutations that alter FKBP12/12.6-RyR interactions promote a Ca^2+^ leak by prolonging the prospect and duration of RyR opening [37,38,39,40,41,42]. Previous TAC tacrolimus research demonstrated that it can spike [Ca^2+^]i-release through its affinity-based binding to FKBP12/12.6 and, subsequently, sequestering it from the FKBP12/12.6-RyR complex, hence elevating its sensitivity to a prolonged [Ca^2+^]i-release [24,43,44] (illustrated in Figure 6B). The current consensus is that upon binding to FKPB12, TAC tacrolimus can sequester this immunophilin from the RyR complex, thereby increasing the sensitivity of this ionic channel to [Ca^2+^]i agonists that may include other targets of IP3R and Ca^2+^ signaling [10,24]. Data obtained in the present study have provided reproducible experimental evidence supporting the latter notion in showing the immediate dissociation of the FKBP12/IP3R complex within their perinuclear and cytosolic locations in the HTR8/SVneo cells upon a transient 1 h exposure to TAC tacrolimus (Figure 5A–E). However, in a recent study investigating the contribution of the IP3R and RyR to the reported altered endothelial function and plausible mechanisms underlying the immunosuppressant-mediated hypertension and endothelial dysfunction in organ transplant patients, Buckley et al. (2020) [45] suggested that the increase in IP3-evoked Ca^2+^ release generated by TAC tacrolimus is mediated by an effect of the drug on calcineurin rather than FKBP modulation itself [45]. Although we acknowledge these findings, nonetheless, we agree with the authors’ suggestion that FK506 may inhibit IP3R by a mechanism that could be independent of either FKBP or calcineurin [45]. This could be true in the face of the fact that TAC tacrolimus binds to other FKBPs, such as FKBP51 and FKBP52, which regulate store-operated Ca^2+^ entry and some TRP channels through steroid receptor signaling to modulate cell function [42,46,47]. Therefore, we believe that our present data are in agreement with previous studies suggesting that TAC tacrolimus exerts its cellular effects through altered Ca^2+^ signaling that can be mediated by displacement of FKBP from IP3R, or through FK506-induced inhibition of calcineurin, or TAC tacrolimus could be indirectly facilitating [Ca^2+^]i release via its bindings to other FKBPs in a variety of cellular contexts. Collectively, our data suggest that TAC tacrolimus can mediate its immune-independent actions in trophoblast cells through influencing [Ca^2+^]i ER release and the concomitant CaAR-induced dynamic global F-actin cytoskeletal rearrangement in treated HTR8/SVneo cells within a relatively short period of time of less than 60 min [29]. Nonetheless, further research into discerning the role of the phosphatase calcineurin in the presently reported TAC-induced [Ca^2+^]i homeostasis in trophoblast cells is warranted.

### 3.2. Selectivity of BAPTA, 2-APB and Related Inhibitors and Chemical Chelators of [Ca^2+^]i

The reported negligible off-target effects of BAPTA, and other related calcium chelating agents used in this study, can validate our current data on the potential IP3R/[Ca^2+^]i-dependent mechanism of action of TAC in human-derived extravillous trophoblast cells [20,23]. Moreover, the reported little to no binding of BAPTA to cell membranes and lack of toxic cellular effects following its intracellular microinjection, can also lend support to validating our present data [20,23]. BAPTA is a relative of the well-known chelator EGTA, in which methylene links between oxygen and nitrogen are replaced by benzene rings, which not only greatly improves it’s selectivity of Ca^2+^ over Mg^2+^ compared to that of EGTA but also improves its rate in Ca^2+^ uptake and release, irrespective of pH changes in the medium [48,49]. Moreover, affinity binding analyses of BAPTA suggest that the presence of quinoline nuclei in their chemical structure was notable for their high sensitivity of fluorescent quantum yields to the binding of Ca^2+^ but not of Mg^2+^ [48]. Furthermore, the reported higher binding affinity and functional selectivity of 2-APB in inhibiting IP3R at a broader concentration range of 10 to 100 μM [21,22] makes it a very useful probe for examining the IP3R agnostic activities of a given compound influencing ER and other intracellular store-operated Ca^2+^ releases in a variety of cellular contexts [42].

### 3.3. Actin Cytoskeletal Organization in Trophoblasts and the Actions of Tacrolimus on Actin Cytoskeleton

Cortical actin filaments undergo constant remodeling that contributes to cellular structure, migration, and division through generating contractile stresses within the F-actin network that gives rise to cortical tension, which determines the overall cell surface mechanics governing cell migration [27,50]. In fact, cortex contractions have been shown to result in the formation of blebs, which function as leading-edge protrusions during cell migration in three-dimensional environments, both in culture and in vivo [51,52,53]. During this process, stress fibers are reportedly distributed throughout the cell in quasi-one-dimensional bundles of actin filaments, which usually connect two adhesion points [26]. Furthermore, most reported molecular mechanisms governing the transitions between these two types of networks dictated subtle crosstalk among actin polymers, a broad library of actins-binding proteins, and contacts with the adhesion molecules integrins and/or cadherins [27]. Studies have also suggested an emerging role for [Ca^2+^]i, in the activation of focal adhesion kinases, mitogen-activated kinase (MAPK), Erk and GTPases, as well as Src family, kinase-based signaling in the remodeling and cytoskeletal dynamics of the cell cortex [27,54]. Based on these reports and our present data, it is reasoned that the TAC tacrolimus-induced formation of actin-rich filopodia in HTR8/SVneo cells indicates a potential for this macrolide in influencing the cellular dynamics of a myriad of actin cross-linkers crucial for severing, capping and recycling of the actin filaments [55,56,57]. Therefore, considering the crucial role of filopodia in cell migration, adhesion, and signal transduction [58], it is reasonable to speculate that TAC can influence cell signaling by modulating intracellular calcium release.

By virtue of their structurally elaborate macrocyclic ring and an extended aliphatic side chain (tail), which terminates with an (E)-N-methyl-N-vinyl formamide (MVF) moiety, actin binding macrolides have the chemical affinity to target and bind actin monomers, thereby potentially influencing the dynamic distribution of the actin cytoskeleton [59,60]. Conspicuously, the macrolactone ring intermingles with a hydrophobic surface on actin subdomain 1, whereas the aliphatic tail encloses onto the tapered hydrophobic cleft that is located between subdomains 1 and 3 [60]. Remarkably, the effects of these barbed end-binding marine macrolides are mimicry of the actions of endogenous actin-binding proteins such as gelsolin, which can impede cell cytoskeleton and filament dynamics, cell motility, and cytotoxicity by splitting and plugging actin polymers [61]. Indeed, several studies reported numerous actin cytoskeleton-related structural defects, comprising dissolution of adherens junctions, cessation of cell–cell and cell–extracellular matrix (ECM) contacts, the demise of cell migration and invasion activity, and arrested cell proliferation expressed in cells exposed to actin-binding macrolides [61,62,63,64]. Unlike this mode of affinity binding reported among actin-binding macrolides, which has the potential of obstructing a large part of the contact interface compulsory for the polymerization of G-actin into F-actin [42,59], our present findings suggest that TAC does not have a similar detrimental effect on F-actin cytoskeletal dynamics. Supportive evidence on the latter has been previously reported by Xiong et al. (2018) [65] on the significant increase in neuronal F-actin/G-actin ratio in pilocarpine-induced status epilepticus (SE) murine model, which is known to be susceptible to progressively deconstructive actin (F-actin) cytoskeletal defects in the hippocampal neurons upon treatment with TAC (FK506) [65]. In understanding the presently reported positive influential cellular cytoskeletal actions of TAC, it is warranted to consider the lack of the (E)-N-methyl-N-vinyl formamide (MVF) terminal moiety in the structure of tacrolimus in lending this stimulatory dynamic actin cytoskeletal influence of TAC. On the other hand, TAC can also promote the stability of F-actin cytoskeleton through its restorative effects on the protein expression and cellular distribution of FKBP12 in a variety of cellular contexts [66]. In their recent studies on the F-actin cytoskeletal restorative actions of TAC in podocytes of a renal injury murine model, Yasuda et al. (2021) [66] reported a podocyte-protective influence of TAC through restoring the protein content and cellular localization of FKBP12 to the actin cytoskeleton in the podocytes of adriamycin-induced renal injury [66]. Intuitively, pondering the proposed regulatory role of the actin cytoskeletal reorganization in determining the fate of trophoblastic stem cells and the specialized function of the various trophoblastic lineages [26], our present findings also suggest a potential use for TAC in therapeutic ART applications aiming at the prevention of transgenerational trophoblast stem cell defects and lineage fate restrictions in subjects in need thereof.

### 3.4. Safety and Pharmacokinetics of Tacrolimus During Pregnancy

Since its discovery in 1984 as a natural macrolactone macrolide produced by the soil bacterium *Streptomyces tsukubaensis*, tacrolimus was first approved by the United States Food and Drug Administration (U.S. FDA) on 8 April 1994 for use in clinical settings requiring immunosuppression [67,68]. Currently, TAC is classified by the FDA as a Class-C drug, potentially unsafe for use during pregnancy [68,69]. This classification was based on an inconclusive, pre-clinical single-dose acute study and a four week oral toxicity study carried out in murine lab animals (rats and mice) receiving 32, 50, 70, 100, 180, and 250 mg/kg (FDA, GLR880181) [69] and 0.32, 1.0, and 3.2 mg/kg (FDA GLR910393) [69], respectively, equivalent to approximately >10 times the required therapeutic levels of TAC for systemic immunosuppression [70]. Despite the FDA currently acknowledging that there is a lack of clinical safety data for the use of TAC during pregnancy [68], several clinical studies across the globe have assessed the safety of TAC for use in pregnant women requiring systemic immunosuppression [71,72,73,74,75]. The currently prescribed clinical dosages of TAC range from 0.075 to 0.3 mg/kg per day, titrated to achieve a trough blood level of 5 to 10 ng/mL in recipients of organ transplants [70]. However, in our pre-clinical experience, and that of others, TAC showed promise in the prevention of implantation failure when administered at much lower dosages of <0.05 mg/kg per day for the first two weeks post-embryo transfer, in women with repeated implantation failure/recurrent early pregnancy loss, with elevated Th1/Th2 cell ratios [7,76], and at a concentration of <10 ng/mL in the murine models of implantation failure in vivo as well as in trophoblast cell cultures in vitro [4,5,9,77,78]. It is imperative to reconcile with our pre-clinical data on the safe use of TAC during pregnancy which are supported by several clinical reports demonstrating that in utero exposure to TAC has no negative influence on fetal growth and development [71,72,73,74,75]. Indeed, data from multiple clinical registries on the use of TAC in pregnant women recipients of organ transplants indicated that the risk of congenital abnormalities is clinically comparable to that found in women who were non-recipients of TAC tacrolimus [71,73,74,75]. These data were also reinforced by a report on the safety of TAC in pregnancy and in utero fetal exposure compiled by Nevers et al. (2014) [79]. Specifically, their report discussing the clinical outcomes released by the National Transplantation Pregnancy Registry (NTPR) in 2012 in pregnant recipients of kidney (n = 334), liver (n = 180), pancreas–kidney (n = 50), heart (n = 45), and small bowel (n = 2) transplants, revealing no evidence of disadvantageous effects for the use of TAC on reproductive health and pregnancy outcomes [79]. Inescapably, the reported clinical pregnancy outcomes narrated by Nevers et al. (2014) dictated 22% to 33% spontaneous abortion, 0% to 2% stillbirth rates, 65% to 74% live birth rates, low birth weight rates of 29% to 53%, and 43% to 72% premature birth rates at a mean gestational age of about 34 to 36 weeks [79].

In this study, TAC was used at the dose of 10 ng/mL based on the safety margin and dose effectiveness reported in previous clinical and pharmacokinetic studies [68,69,70]. TAC is a substrate for P-glycoprotein, which is among several drug transporters expressed and active on placental syncytiotrophoblasts [80], which helps minimize fetal exposure by a backward effluxing drug into the maternal circulation [81]. Accumulating evidence from clinical studies revealed that the concentration of TAC tacrolimus in whole blood does not broadly reflect its concentration at the intracellular site of action for calcineurin inhibition [81,82]. In their recently released retrospective cohort study on modeling changes in the pharmacokinetics of TAC during pregnancies that occurred after kidney transplantation, Schagen et al. (2023) [83] dictated that TAC clearance increases during pregnancy, resulting in decreased exposure to TAC [83]. They derived this finding by compiling a total of 260 whole-blood TAC at pre-dose concentrations from 14 pregnant kidney transplant recipients. In plasma, TAC binds to the α1-acid glycoprotein and albumin [84,85], thus leaving the unbound drug available to binding receptors and cross membranes, including those involved in transplacental drug transfers [86].

Indeed, differential protein binding between fetal and maternal circulations has long been recognized as a critical determinant of placental drug transfer since it is the unbound drug that equilibrates across the placenta [87,88]. Clinical data reported a low percentage of unbound TAC in plasma (5.4 ± 0.7% during mid- and late pregnancy and 2.8 ± 0.4% in maternal plasma post-partum) [86]. Moreover, the blood plasma ratio ranging from 4 to 42 TAC concentrates in erythrocytes, which was reported by Hebert et al. (2013) [86] was, in fact, believed to be due to a lower unbound fraction of TAC in whole blood during mid- to late gestation [86]. These data were also echoed by the pharmacokinetic studies on TAC blood concentrations during pregnancy reported by Zheng et al. (2013) [89], which showed that a 91% and 100% increase in TAC-free fractions in plasma and blood during pregnancy, respectively, were inversely correlated with both hematocrit and red blood cell counts [89]. These findings also suggest that the binding of tacrolimus to erythrocytes restricted its availability for metabolism and necessitated the need for increased TAC dosages during pregnancy by an average of 45% to maintain systemic immunosuppression [89]. Notably, these pregnancy-associated alterations in the free fractions of TAC were inversely associated with serum albumin concentrations, which decreased by 27% during pregnancy thereby significantly contributed to the observed gestational increase in the TAC whole-blood concentrations reported in these clinical studies [89]. Consequently, the current clinical consensus dictates that pregnancy-related alterations in TAC pharmacokinetics require constant dose adjustments to maintain a whole-blood TAC concentration in the desirable therapeutic range during pregnancy conducive to achieving systemic immunosuppression [86,90]. Conspicuously, the concerns raised by these clinical data on the plausible impact of increases in freely circulating TAC concentrations during pregnancy on clinical outcomes warrant the need for alternative therapeutic approaches aimed at a localized drug delivery of low-dose TAC in treating implantation failure and early pregnancy loss in women in need thereof.

## 4. Materials and Methods

### 4.1. Cell Lines and Tacrolimus Dose Formulation

The immortalized, human-derived, first trimester, extravillous trophoblast (EVT) cells, HTR8/SVneo, generated from first-trimester human placenta, were generously provided by Dr. Charles Graham of Queen’s University, Kingston, ON, Canada. Briefly, HTR8/SVneo cells (passages 76–83) were cultured in a RPMI-1640 medium containing L-glutamine and sodium bicarbonate (Cat# R8758, Millipore Sigma, Oakville, ON, Canada), supplemented with a 5% fetal bovine serum (Cat# F2442, Millipore Sigma) and a 1% antibiotic antimycotic solution (Cat# A5955-100ML, Millipore Sigma). Tacrolimus (TAC, FK506) (10 ng/mL) (Cat# B415260, Toronto Research Chemicals, Toronto, ON, Canada) was dissolved in dimethyl sulfoxide (DMSO) HybriMax^TM^ (Cat# D2650, Millipore Sigma) and administered to each culture according to an established protocol [9].

### 4.2. Intracellular [Ca^2+]^ Detection

The cell imaging with a two-photon confocal microscope was performed at the Queen’s Cardiovascular and Pulmonary Unit (QCPU). In brief, the effect of TAC on intracellular Ca^2+^ release in live HTR8/SVneo cells, cultured on 35 mm glass bottom Petri dishes, was monitored using the cell-permeable Ca^2+^ fluorescent marker Fluo-4 AM (Cat# F14201, Invitrogen, Burlington, ON, Canada) and a two-photon confocal microscope (Leica TCS SP8 w/WLL & HyVolution & InSight DS, Concord, ON, Canada) available at the QCPU. To decipher the mode of action of the TAC in these cells, Ca^2+^-dependent IP3R signaling was inhibited in live HTR8/SVneo using the phospholipase C (PLC) inhibitor, U73122 (10 μM; Cat# U6756, Millipore Sigma) and its inert derivative, U73343 (10 μM; Cat# U6881, Millipore Sigma), the membrane-permeable modulator/inhibitor of the intracellular, IP_3_-induced, calcium release of 2-Aminoethyl diphenylborinate (2-APB, 100 μM; Cat# A609115, Toronto Research Chemicals), the PI3K inhibitor Wortmannin (10 μM; Cat# W1628, Millipore Sigma), and the highly-selective Ca^2+^-chelating agent BAPTA (10 μM; (Cat# B123500, Toronto Research Chemicals). The individual plates of cell cultures were incubated with 4 µL of Fluo-4 for 30 min prior to the start of imaging. Prior to treatment, a baseline measurement of fluorescence was obtained for a sustained period of 5 min tracing, after which either TAC or an inhibitor of the IP3R signaling pathway was administered. For cells only treated with TAC, the recording of fluorescence continued for another 5 min. For cells treated with an inhibitor, the recording continued for 3 min or 5 min before the addition of TAC. Following the addition of TAC, recording continued for an additional 6 min. The selection and molar concentrations of the various inhibitors reported in this study were determined based on published studies, suggesting the limited off-target effects of these inhibitors of intracellular Ca^2+^ release and chelating agents [42,91,92].

### 4.3. F-Actin Labeling in Live HTR8/SVneo Cells

To investigate the effect of TAC on the dynamic distribution of F-actin within the cells, the CellMask Green^TM^ actin tracking stain (Cat# A57243, Invitrogen) was used to detect F-actin in live cells. The CellMask Green^TM^ actin labeling stain readily passes through live cell membranes and is preserved within live cells after loading, and there have been no reported detrimental effects on the viability of various cell lines after 24 h of incubation. Notably, CellMask Green^TM^ does not label globular actin (G-actin) or monomer actin (M-actin). Briefly, HTR8/SVneo cells were incubated with 1× diluted CellMask Green^TM^ for 30 min at 37 °C and 5% CO_2_, according to the manufacturer’s instructions. Nuclei were counterstained with DAPI prior to rinsing the cells 4× in a wash buffer at 37 °C and images were acquired using a two-photon confocal microscope, which was performed at the Queen’s Cardiovascular and Pulmonary Unit (QCPU).

### 4.4. Live-Cell Imaging for F-Actin

Live HTR8/SVneo cells were pre-incubated with a CellMask Green^TM^ actin tracking stain for 30 min prior to image acquisition using the OkoLab stage-top microscope incubator (OkoLab Bold Line, Pozzuoli, Italy) live imaging system connected to a Leica TCS SP8 confocal microscope (Leica Microsystems, Wetzlar, Germany). 3D Images (z = 24 stacks) were taken using the objective HC PL APO CS2 63×/1.40 oil (zoom 4.5; pixel size 39.73 nm), the white-light laser tuned on 499 nm, and a HyD detector (504–581 nm). Four different fields were aleatorily imaged by a scientist blinded to the treatments (n = four per treatment).

### 4.5. Co-Localization Analysis of IP3R and FKBP12 in HTR8/SVneo Cells

To test the hypothesized role of TAC in spiking intracellular Ca^2+^ [Ca^2+^]i via its interaction with the immunophilin binding protein FKBP12, and the subsequent structural destabilization of the RyRs [43], 50% confluent monolayers of the vehicle (DMSO)-treated (untreated control) and TAC-treated (1 h treatment) HTR8/SVneo cell, previously cultured on sterile glass slides in six-well plates and overnight starved in the FBS-free RPMI-1640 medium (Cat# R8758, Millipore Sigma) containing 0.05% DMSO, were fixed in 4% paraformaldehyde in PBS for 15 min at room temperature. Cell membranes were subsequently permeated using a PBS-based permeation buffer containing 0.1% Tritton X-100 for 20 min, prior to the 1 h incubation, at room temperature in a blocking buffer solution with a 5% fetal calf serum in PBS containing 0.05% Tween-20 (PBST), following an established protocol [11]. Incubation with Alexa Fluor 680-conjugated anti-IP3R-1 (Cat# sc-271197 AF680, Santa Cruz Biotech. Inc., Dallas, TX, USA) or Alexa Fluor 790-conjugated anti-FKBP12 (Cat# sc-133067 AF790, Santa Cruz Biotech. Inc.) primary antibodies were carried out at 4 °C overnight in a dark humidified chamber. Due to current technical limitations with the wavelength detection of AF790 fluorochrome, monolayers of HTR8/SVneo cells were first incubated with Alexa Fluor 790-conjugated anti-FKBP12, rinsed in three changes in a PBST buffer and incubated with a FITC-conjugated goat anti-mouse secondary antibody for 1 h at room temperature to allow for affinity antibody binding for the immunofluorescent detection of FKBP12 in these cells. Subsequently, cells were rinsed thrice in PBST and similarly incubated overnight with Alexa Fluor 680-conjugated anti-IP3R-1 in a humidified dark chamber at 4 °C. Residual primary antibody suspensions were then cleared with three changes in PBST, and nuclei were counterstained with 4′, 6-diamidino-2-phenylindole (DAPI) (Thermo-Scientific Fisher, Ottawa, ON, Canada). The images were captured using a TCS SP8 laser scanning confocal microscope (SP8 Leica, Concord, ON, Canada) equipped with the HC PL APO CS2 63×/1.40 oil objective, set at a zoom of three to four (resulting in a pixel size of 36 nm to 53 nm). The laser settings used were: 405 nm (at 28% power; DAPI), 495 nm (at 25% power; FITC), and 670 nm (at 20% power; Alexa-Fluor 680). Z-stack imaging was performed, and the image with the best focal plane was selected for quantifying the mean fluorescence intensity (MFI) and measuring Pearson’s correlation. The experimental controls that were used to ensure the specificity of the immunofluorescence included: (1) unstained, (2) single-stained with IP3R-I-Alexa-Fluor 680, (3) single-stained with FKB12-Alexa-Fluor 790, and (4) single-stained with anti-mouse-FITC. The LAS X Life Science Microscope Software Platform LAS-X software (LASX Office 1.4.7 28921)was employed for image acquisition and analyses.

### 4.6. Western Blot Analysis

The influence of a 1 h treatment with TAC on the protein expression of IP3R (IP3R-1) and FKBP12 in HTR-8/SVneo cells was detected by Western blotting. Protein concentrations were determined by using the Bradford protein assay followed by an incubation of the Western blot membranes containing a 15 μg protein/sample of HTR8/SVneo cell lysates, with an Alexa Fluor 680-conjugated anti-IP3R-1 (Cat# sc-271197 AF680) or an Alexa Fluor 790-conjugated anti-FKBP12 (Cat# sc-133067 AF790) for the detection of their respective proteins in these cells. In brief, cells were separated into aliquots containing 15 μg of protein and mixed with 5 μL of 6× SDS loading buffers. Samples were run on 10% and 7.5% polyacrylamide gels, and blots were transferred onto PVD membranes. One hour incubation in 5% non-fat milk was applied to block the non-specific binding of the detection antibodies, and blots were subsequently probed with 1:200 dilutions of the respective primary antibody for the exposure and analysis of band intensities using the Odyssey^®^ DLx Infrared Imaging System (LI-COR Biotech. LLC., Lincoln, NE, USA) and the Empiriastudio Image Analysis software (Version 2.3.0.154 (2.3.0.154). α-tubulin was used as a comparative control for all Western blot analyses carried out in this study.

### 4.7. Statistical Analyses

GraphPad Prism (Version 10.0) software (GraphPad Software, San Diego, CA, USA) and StatPlus (https://www.analystsoft.com/en/, (accessed on 1 April 2022)) were used for all statistical analyses. The normality of data distributions was validated using the Kolmogorov–Smirnov method and parameters of normally-distributed data were presented as Mean ± S.D. A one-way ANOVA followed by Neuman–Keuls, Fisher LSD, and Scheffe’s ad hoc were used for pairwise comparisons of the independent parameters. A *p*-value of less than 0.05 was considered statistically significant.

## 5. Conclusions

The findings in the present study demonstrated that tacrolimus (TAC) could mimic a [Ca^2+^] ionophore with a relatively high potency, as determined by fluorescent live-cell imaging using Fluo-4. Intriguingly, unlike the use of Wortmannin, TAC was unable to recover [Ca^2+^]i in the presence of the selective IP3R inhibitor, 2-APB. Therefore, this receptor may play an important role in TAC mechanism of action. Moreover, we have demonstrated that TAC has the potential to influence the migration and invasion of the trophoblast through downstream [Ca^2+^]i-mediated intracellular events and pathways involved in trophoblast migration and invasion, namely F-actin cytoskeletal redistribution. Further research into the mono-therapeutic uses of TAC supporting local and targeted drug delivery to selectively promoting trophoblast migration. In clinical settings requiring in vitro fertilization and embryo transfer is warranted

### Study Limitations

The use of the HTR8/SVneo cell line.

The reported existence of stromal/mesenchymal cells within monolayers of HTR-8/SVneo cells signifies a main limitation of this study. The reported heterogeneity of the HTR-8/SVneo cell cultures is believed to be due to the presence of mixed populations of the so-called “side populations” of immature vimentin-positive cells, including stem cells and progenitor cells that originate from either the purification and generation of HTR-8/SVneo or from an ongoing epithelial-to-mesenchymal transition (EMT) process [93,94]. Future investigations should consider the use of primary first-trimester extravillous trophoblast cells for a comparison.

## Data Availability

Due to institutional guidelines on the sharing of research information, the data presented in this study are available on request from the corresponding author.
